# Development of a Survivorship Care Plan (SCP) Program for Rural Latina Breast Cancer Patients: *Proyecto Mariposa—*Application of Intervention Mapping

**DOI:** 10.3390/ijerph17165784

**Published:** 2020-08-10

**Authors:** Eunjeong Ko, María Luisa Zúñiga, Susan I. Woodruff, Yolanda Serra-Martinez, Veronica Cardenas

**Affiliations:** 1 School of Social Work, San Diego State University, 5500 Campanile Drive, San Diego, CA 92182-4119, USA; mlzuniga@sdsu.edu (M.L.Z.); swoodruff@sdsu.edu (S.I.W.); 2 School of Public Health, San Diego State University, 5500 Campanile Drive, San Diego, CA 92182-4119, USA; yserramartinez@sdsu.edu; 3 Moores Cancer Center, 3855 Health Sciences Dr. La Jolla, University of California, San Diego, CA 92093-0658, USA; vcardenas@health.ucsd.edu

**Keywords:** survivorship care planning, breast cancer, rural Latinas, intervention mapping

## Abstract

Latina breast cancer survivors are less likely to receive a comprehensive Survivorship Care Plan (SCP) than non-Latina Whites. Evidence-based and theory driven interventions are needed to promote a culturally and linguistically responsive SCP. This paper describes the application of Intervention Mapping (IM) in the development of *Proyecto Mariposa*, a comprehensive SCP program targeting Latina breast cancer survivors living in a rural U.S.–Mexico border region. We conducted a needs assessment using focus groups (*n* = 40) and individual interviews (*n* = 4) with stakeholders to elicit their needs and preferences relating to SCPs and SCP aid (Step1). Content analysis of transcripts was conducted using Atlas.ti. The findings informed the development of a matrix of change objectives where we selected specific behavioral theories to ground the practical application of the SCP program (Step 2). We identified behavioral theories and the practical application of behavioral change (Step 3) and designed and developed a comprehensive SCP program which consisted of a culturally—and linguistically—adapted SCP document and animated video as an SCP aid (Step 4). The systematic application of the IM framework resulted in the development of a comprehensive and culturally tailored SCP intervention. Stakeholder active involvement in the cultural tailoring of the program was imperative and strengthens the SCP intervention.

## 1. Introduction

Breast cancer patients in the U.S. comprise the majority of cancer survivors (about 23%) [1]. Cancer survivors face challenges during the complex extended survivorship phase, including physical, psychological, financial, and spiritual challenges [2]. These challenges are of special importance among Latinas who, compared to non-Hispanic Whites, are more likely to be diagnosed at an advanced cancer stage [3]. Latina breast cancer survivors have lower survivorship knowledge and greater dissatisfaction with care information [4], unmet physical symptom management, lack of social support, and need for formal transition to follow-up care compared to non-Latina counterparts [5]. 

In recognition of needed support during the transition to the survivorship phase, the Institute of Medicine (IOM) recommends that upon completing treatment, cancer patients receive a comprehensive treatment summary and follow up care plans, often called a Survivorship Care Plan (SCP) [6]. Nevertheless, since the IOM recommendation in 2005 [6], only 20% of cancer care providers report always or almost always providing SCPs to patients [7]. Demographic characteristics such as race/ethnicity, socio-economic status, and geographic factors may play a role in whether individuals receive an SCP. For example, rural patients may be less likely to receive an SCP than urban patients [8] and face challenges to an optimal transition to cancer survivorship due to disproportionately low access to health care resources, low socioeconomic status, and language barriers [9,10,11]. In addition, Latino cancer patients living in the U.S.–Mexico border region frequently seek health care on both sides of the border, which complicates care coordination [12,13]. 

Intervention development and evaluation studies with Latina breast cancer survivors are needed, especially among rural Latinas where research into the unique needs for survivorship care is especially lacking. Ashing and colleagues used a community-based approach to develop a much-needed Spanish language SCP template [14] for urban-dwelling Latinas. Additional work is needed in the SCP field to address the needs of rural low-income Latinas who may have unique linguistic, cultural, geographic, and system-level challenges to their care continuity. Culturally tailored interventions are important for reducing health disparities, yet little is known about the needs of rural Latina breast cancer patients or the effectiveness of SCP for such patients. Addressing survivors’ various concerns and unmet needs by using an SCP document alone is likely insufficient [15,16,17]; moreover, cancer organizations have suggested developing an effective tool kit (aids) to supplement the SCP and tailor information to the specific needs and geographic location of the patient [16]. Thus, the purpose of the present study is to describe the development of a culturally appropriate, theory driven program, called *Proyecto Mariposa* (Butterfly Project) for Latina breast cancer survivors living along the U.S. side of the California and Baja California (Mexico) border. *Proyecto Mariposa* included an adapted SCP document and supplemental aid.

In the current study we describe the development of *Proyecto Mariposa* within the context of Intervention Mapping (IM), a systematic, theory-based approach to design, create, and implement public health programs and corresponding interventions [18]. IM includes community-based participatory research in order to be responsive to the priorities and needs of the communities for whom the interventions are intended [19]. To this effect, we undertook the formative research for *Proyecto Mariposa* with the full participation of a rural community-based cancer organization, a cancer clinic, and input from rural Latina breast cancer survivors, their family caregivers, and clinical care team members.

## 2. Methods

The full spectrum of Intervention Mapping spans six different steps, the first four are related to the development phase (i.e., assessing community needs, creating a matrix of change objectives, application of theory and practical application, and production of the intervention), and the last two relate to the implementation and evaluation phase. This paper focuses on the development phase of the *Proyecto Mariposa* SCP program (Steps 1–4) described in Table 1 where we depict the actions taken in each of the four development steps.

### 2.1. Step One: Needs Assessment

The first step in our IM process focused on identifying the survivorship care planning needs of Latina breast cancer survivors and the contributing factors for their active utilization of an SCP. This step involved articulating the health problem, target population, and determinants of the problem. We conducted a needs assessment using two approaches: (1) a comprehensive literature review of SCP among Latina breast cancer patients relating to patient challenges and needs for survivorship care and (2) formative qualitative research assessing determinants to utilize SCP and preferences regarding SCP layout and presentation.

#### 2.1.1. Participants and Procedure

The setting in which this study took place is a rural area, and vastly medically underserved region located in Southern California on the U.S.–Mexico border. This area’s cancer-related mortality rates are similar to statewide rates [20], but there are only two medical oncology clinics and one radiation clinic in the 4500 square mile county.

We conducted a qualitative study using focus groups and individual interviews with stakeholders. These included eight focus groups (*n* = 40) comprised of: breast cancer survivors (2 groups), family caregivers (2 groups), nurses (2 groups), medical assistants (1 group), and social work patient navigators (1 group). Individual interviews were conducted with four physicians (two oncologists and two primary care physicians). The interview guides focused on (a) participant preferences and opinions regarding types of SCP materials and layout, (b) preference for an SCP visual aid to facilitate understanding and relevance of the SCP (e.g., *fotonovela*, animated video, or pamphlet), and (c) other cultural considerations regarding framing of SCP messaging. From the focus groups with health care providers, we also elicited recommendations on how to optimize the clinician and patient navigator collaboration in SCP implementation.

Research team members conducted the focus groups in Spanish or English and sessions lasted up to one hour. All focus groups and individual interviews were audio-taped with participant consent and then transcribed and translated into Spanish by the research assistant. This study implemented a formative approach to develop an intervention program. The San Diego State University Institutional Review Board (IRB) reviewed and granted exemption from full IRB review. Although the study was deemed exempt, investigators chose to include a voluntary and informed consent procedure as a means to further support ethical intent for participants in their decision to participate or not. San Diego State University IRB reviewed the consent form.

#### 2.1.2. Data Analysis of the Formative Focus Group Research

Data were analyzed using thematic analysis [21]. Our *a priori* themes included: (1) personal determinants (e.g., knowledge) and environmental factors (e.g., clinic staffing) and (2) preferences for SCP document and SCP aid. The research team reviewed all transcripts and developed and achieved a consensus on the initial coding and revised the codes as needed to achieve the final coding scheme. All research team members then independently hand coded the data. Data were later entered into Atlas.ti (v.8) [22] to facilitate the retrieval of coded text for the selection of representative quotes. Findings were then used to guide subsequent development of the SCP document and aid as we describe in the results.

### 2.2. Step Two: Specifying Program Outcomes and Creating a Matrix of Change Objectives

This step described the specific desired program outcomes (e.g., behavioral, environmental), articulated the mechanism or process that was to be targeted (i.e., performance objectives), and how it would be targeted to promote the desired outcome. We then created a matrix of the program outcome by performance objectives and determinants. Who will and what is needed to achieve performance objectives for each determinant were also specified in the Matrix of Change Objectives.

### 2.3. Step Three: Selecting Theory-Based Methods and Design Practical Applications

We designed the program using behavioral theories that underlie the methods and practice application for behavior change. The application of theoretical constructs is important because it helps to understand and guide practical application to successfully deliver the intervention [18]. These theoretical constructs guide us to better understand the issues related to patients’ mechanisms for SCP use, optimize factors that promote behavior change, and further suggest solutions and outcomes [18]. In this step, theory and evidence-based methods were selected in response to determinants and performance objectives.

### 2.4. Step Four: Program Production

Program production involved designing and creating the *Proyecto Mariposa* program by drafting SCP materials and refining a manual on how the SCP intervention would be delivered. The SCP and animated video development were responsive to cultural preferences expressed by the breast cancer survivors and their families, embodying elements of cultural tailoring that are necessary to develop culturally effective interventions [23]. Step 4 builds on Steps 1–3 where we developed and culturally-tailored the intervention based on participant stated preferences and recommendations. Program production included conducting confirmatory focus groups with the same stakeholders from the first round of focus groups. A draft of the SCP document and a storyboard for the animated video were presented in the focus groups eliciting final feedback and suggestions prior to finalizing the intervention production. Participant recommendations were summarized and implemented to improve the SCP document and video. Once the video script was completed, we conducted a readability test using INFLESZ (v1.0). [24] to promote patient comprehension of the content.

## 3. Result

### 3.1. Step One: Needs Assessment

The literature review on breast cancer survivorship and its determinants indicated significant unmet survivorship care needs among Latina breast cancer survivors including issues related to finance, symptom management, and survivorship care related information [4,25] and poor mental and physical health and low quality of life [26,27,28]. The determinants for the optimal use of SCP included knowledge about survivorship related issues [4,29,30], psychological distress (i.e., anxiety and fear concerning cancer recurrence) [26,27,31], and lack of confidence in patient–physician communication and management of chronic illness [30,32,33].

#### Results from the Focus Groups

Themes analyzed from the qualitative data were organized by (1) personal determinants and environmental factors and (2) preferences for SCP and SCP aid.

**Personal Determinants and Environmental Factors**. Formative research results yielded similarities with findings from the SCP literature; however, our study revealed different contextual, social–economic, and geographic features and cultural characteristics that are important to this population of rural Latina breast cancer survivors and their care team (Table 2). Themes that emerged from the focus groups included survivors’ (1) lack of knowledge about cancer information and low health literacy (e.g., medication names, dates and types of surgery, medical terminology), (2) difficulty retaining information that was provided during clinical encounters (e.g., language barriers and low health literacy), (3) lack of proactive behavior during the clinical encounter (e.g., reliance on physicians and low self-efficacy to ask questions of physicians), (4) psychological distress (i.e., fear of test results and words associated with cancer), and (5) structural barriers including transportation, lack of care coordination, lack of staff linguistic competence, and lack of financial resources. Earlier studies of environmental and structural determinants impacting the implementation of SCP included shortage of staff, time constraints, and lack of resources [29,34] which were similar to our focus group findings.

**Preferences for SCP and SCP Aid**. Participants in all groups overwhelmingly supported the use of patient navigators to provide educational information about the SCP document. SCP was seen as a tool for (1) providing a good source of information for improving survivorship care and (2) facilitating patient–provider communication on a survivorship care issue. In regard to the types of SCP aid, stakeholders favored an animated video to impart information about SCP and strategies to promote greater patient self-efficacy when discussing their SCP document and questions with providers. In sum, our qualitative research resulted in the inclusion of two key intervention components: a culturally-tailored and bilingual (Spanish–English) breast cancer SCP and a program aid in the form of a Spanish-language animated video.

In terms of format and person to deliver SCP, stakeholders preferred the SCP in a paper format delivered by the nurse, followed by an educational session conducted by the social work patient navigator. Specifically, the social work patient navigator role was to: (1) reinforce the SCP education provided by the nurse, (2) provide behavioral coaching for proactive behavior (i.e., use the SCP for asking questions to their physicians), and (3) provide instrumental support (e.g., counseling on insurance conversion, transportation arrangement, etc.). The results of stakeholders’ preferences and suggestions for SCP and SCP aid are reported in detail in Step 4.

### 3.2. Step Two: Specifying Program Outcomes and Creating a Matrix of Program Objectives

In this step, we specified the target population and described our overall program outcome: Rural Latina breast cancer survivors age 21 years and older will actively utilize their Breast Cancer SCP (i.e., review SCP for their cancer treatment related information and use of SCP to ask questions to clinicians) for their survivorship care. Performance objectives were determined by specifying what Latina breast cancer survivors needed to perform or an action they could take (e.g., share their SCP with physician and family members and using the SCP for asking questions to physicians) in order to achieve the program outcome (i.e., improved survivorship planning). The actions for performance objectives were described for each determinant including cognitive factors and skills such as knowledge, intention, self-efficacy/skills, attitudes, motivation, and outcome expectations (see Table 3).

### 3.3. Step Three: Selecting Theory-Based Methods and Design Practical Applications

The two main methods we selected included: entertainment education and navigator-led patient activation. Entertainment education [35] incorporates important health messages into a story-telling format which targets change determinants using additional behavioral methods including (1) a reinforcement method from Social Cognitive Theory [36], (2) tailoring to stage of change from the Transtheoretical Model [37], and (3) modeling from Social Cognitive Theory [36]. Strategies to operationalize these methods included the patient navigator showing an animated video to the patient, emphasizing the importance for the patient to review her SCP to gain knowledge about her medical information and follow recommendations in the SCP, and increasing patient self-efficacy to discuss with her providers issues relating to her needs and concerns about survivorship care (reinforcement method from Social Cognitive Theory) [36]. Patient navigators also reinforce patient behavior by demonstrating effective survivor–provider communication via role-play (modeling from Social Cognitive Theory) [36]. Communication strategies implemented by the navigators are tailored to the individuals’ stage of motivation for behavioral change (tailored to stage of change from the Transtheoretical Model) [37]. This will lead to patient activation [38] by promoting individual willingness and ability to engage in action for the desired health behavior via modeling where a model coaches and demonstrates target behaviors to increase patient self-efficacy and reinforce patient motivation.

### 3.4. Step Four: Program Production

*Proyecto Mariposa* consists of two components: an adapted breast cancer survivorship care plan (SCP) and an SCP aid (i.e., an animated video).

#### 3.4.1. SCP Adaptation

We identified commonly used existing SCP documents from American Society of Clinical Oncology (ASCO) [39] and the Journey Forward program [40] and compared the relevance and applicability of these SCPs for our target population. We then presented these existing SCP documents to *Proyecto Mariposa* stakeholders for feedback. ASCO Breast Cancer SCP was selected by the stakeholders for the development of the Spanish-language version of the survivorship care plan. Informed by our qualitative research, we made linguistic and cultural adaptations to the ASCO SCP in the following areas: language, format, color, and content. **SCP Language**: Bilingual (English–Spanish) researchers and a bilingual/bicultural consultant undertook an iterative process of translation and review to ensure the accuracy and linguistic relevance of translation, paying particular attention to wording and phrases suitable for Mexican/Mexican Americans. **SCP Format and Content**: Through collaboration with a media graphic specialist with expertise in Latino audiences, we formatted the SCP document graphics and symbols to improve document readability and patient comprehension. Following input from stakeholders, we added target population specific issues (i.e., transportation issue in rural regions), a list of cancer resources at the local, national, and international level (i.e., some survivors utilize health care both in US and Mexico), as well as space in the document for survivors to note additional treatment specific content (i.e., dates of radiation therapy treatments).

#### 3.4.2. Drafting and Developing SCP Aid (i.e., Animated Video)

Stakeholders preferred an animated video as an SCP aid. Informed by our focus groups, we drafted the script of the animated video with a tailored message, focusing on promoting cognitive functioning (i.e., increasing knowledge about SCP related information), positive affective change (enhancing self-efficacy and empowerment), and behavioral functioning (i.e., activating their proactive behaviors). We undertook the following cultural tailoring activities to enhance the cultural appropriateness of the animated video. First, animation characters were created reflecting sensitivity to characteristics that may be shared among Latina breast cancer survivors including age (i.e., middle age), hair length (i.e., short or loss of hair due to cancer treatment after chemotherapy), and consideration of traditional gender roles (e.g., family relationships; daughter as a caregiver). Second, script messages were developed considering binational lives and care-seeking practices both in the US and Mexico. For example, we included a statement in the script that suggested sharing their SCP with their physicians and family who may be involved with the participant’s survivorship care in Mexico. Third, we tailored messages that took into consideration health literacy and potential low educational attainment of some members of the target population. For example, we used lay medical terminology (i.e., high blood pressure vs. hypertension). The research team members and a bilingual/ bicultural media consultant applied an iterative process of review and editing of the video script to ensure linguistic and cultural appropriateness. We tested the readability of the script using INFLESZ v1.0 [24], which found that the script was written at the 4th grade level. Prior to finalizing the video production, we conducted confirmatory focus groups with the same stakeholders from the first round of focus groups. In these sessions, we showed a draft of the SCP document and a storyboard for the animated video. Participant recommendations were summarized and incorporated to improve the SCP document and video.

## 4. Discussion

This article describes the application of an intervention mapping approach (development phases: Step 1 to Step 4) to develop a theory-based breast cancer SCP and educational aid for rural Latina breast cancer survivors. There was participant consensus that SCP plays an important role in filling patient information gaps and guiding patients on improved communication with their clinicians (i.e., asking question) for their survivorship care. Preferences for the SCP format (e.g., print vs. web-based) may vary by age group; however, in our breast cancer survivor focus groups, we did not find differences in preferences by age (M = 51.7; ranges 28–67 years). The SCP can provide a clinically useful summary and communication tool for both the patient and clinicians with whom the patient interacts. Stakeholder input deepened our understanding about socio-cultural and structural challenges survivors encountered and their feedback and insights directly informed the development of the SCP document itself and the educational video. A previous study with Latino cancer survivors [41] found that addressing cultural values and concepts (e.g., *familismo*) is vital to the development of a culturally effective intervention. Cultural adaptation that is responsive to a border setting is imperative for these Latina breast cancer survivors, given that they hold strong cultural values and beliefs, and many lead binational lives. Although we did not develop the SCP intervention specifically for binational use, the availability of the SCP document in Spanish can perhaps facilitate patient sharing of clinical information with their clinicians in Mexico and thereby facilitate improved continuity of care.

Through the application of entertainment education in the form of an educational video, *Proyecto Mariposa* employed a theoretically driven approach to its mass communication strategy to influence Latina breast cancer survivors’ social and behavioral change to improve survivorship planning. Culturally tailored health messages with graphics and images such as those we used in *Proyecto Mariposa* is particularly beneficial for individuals with low health literacy [42,43]. Our video included culturally relevant and sensitive characters and storylines to which many Latina viewers could relate. This tailoring enhances the likelihood that the target population will successfully engage with this educational entertainment [44].

*Proyecto Mariposa* capitalizes on the important role of the patient navigator to engage the patient in their care. Navigator-led patient activation is a key behavioral method which builds patient confidence and ability to manage their health and health care, especially among underserved cancer patients [45,46]. For example, our work supported proactive behavior, an area that prior research has identified as challenging for some Latinas who have low self-efficacy regarding patient–provider communication [47]. Our intervention is designed to facilitate patient-navigators’ support of patient self-efficacy to openly discuss difficulties and needs about their survivorship care with their providers. Patient navigators are in a unique position to go beyond traditional patient navigation activities by facilitating patient-centered care and responding to patients’ needs for information and coordination of community resources in a timely manner [45,46]. Supplemental aids used by the patient navigator (e.g., animated video) could provide not only information but also a model of the elements of activation through its characters which will result in producing better cancer outcomes for patients including ethnic/racial minorities and low-income patients.

### Limitations

This study has important limitations that bear reflection. This study is formative, qualitative research with a limited sample size that may not reflect the survivorship care needs and educational preferences of other Latina populations in other geographic regions. Nevertheless, this study is one of few theory-based interventions that embraced the IM process, including continuous feedback loops with survivors and health care providers. Although we planned and approached SCP document adaption systematically eliciting stakeholders’ input, we cannot say with certainty to what extent the adaption is sound until it is tested. Our work expands the knowledge on culturally tailoring an SCP intervention. *Proyecto Mariposa* will be implemented following Step 5, and the evaluation of program (Step 6) will be reported in the subsequent paper upon completing the intervention.

## 5. Conclusions

IM provided a clear and reproducible approach to identify specific steps in the behavioral intervention process, with the flexibility to be fully responsive to and meet the needs of our target population and community. The cultural adaption of an intervention is a complex process involving addition, deletion, or modification of intervention components to best meet the needs of the target population. The process we undertook, and the detailed documentation we described on the development and cultural tailoring of this SCP intervention for rural Latina breast cancer survivors expands the knowledge base on key factors that clinicians should consider to better serve Latinas in their breast cancer survivorship, particularly those who may receive health care from different health systems, such is the case in a binational, U.S.–Mexico border context. *Proyecto Mariposa* will be implemented and undergo evaluation to complete the IM process, and findings will be reported in a subsequent paper.

## Figures and Tables

**Table 1:** Steps and Activities for the Development of *Proyecto Mariposa*.

Step	Specific Activities
Step 1: Logic Model of the Problem	1. Established and developed a planning group that includes researchers and stakeholders 2. Conducted a breast cancer survivor needs assessment including a literature review and focus groups 3. Described the context for the intervention including population, setting, and community 4. Identified determinants 5. Stated program goals
Step 2: Program Outcomes and Objectives; Logic Model of Change	1. Articulated expected outcomes for patient behavior and clinical environment 2. Specified performance objectives for these outcomes 3. Identified determinates for behavioral and environmental outcomes 4. Constructed matrix of change objectives
Step 3: Program Design	1. Selected theories and evidence-based change methods 2. Designed the practical application to deliver our change method 3. Generated program themes, components, scope, and sequence
Step 4: Program Production	1. Applied findings from the qualitative data to inform program structure and organization 2. Prepared plans for program materials 3. Drafted messages, materials, and training manual 4. Refined and produced materials

**Table 2:** Determinants Impacting the Use of Survivorship Care Plan (SCP).

Categories	Subcategories
**Personal determinants**	**Cognitive factors**
	Knowledge about survivorship care related issues
	Low perceived risk for not utilizing SCP
	Low health literacy
	**Affective factors**
	Fear of cancer recurrence/anxiety
	Depression
	Feeling overwhelmed
	Lack of motivation
**Environmental determinants**	**Organization/system barriers**
	Lack of staff members
	Lack of time
	Insufficient resources
	No standardized practice guideline
	**Structural factors**
	Lack of staff linguistic competence
	Lack of transportation
	Lack of community resources (i.e., medical resources, supportive care)

**Table 3:** Sample Cells from Matrix of Change Objectives.

Behavioral Outcome: Rural Latina Breast Cancer Survivors Will Actively Utilize SCP for their Survivorship Care					
Performance Objectives (PO)	Determinants				
	Knowledge (K)	Intention(s) (I)	Self-Efficacy/Skills (SE)	Attitudes (A)	Outcome Expectations (OE)
**PO.3. Survivors will use SCP for communications with health care provider (HCP)**	K.3. Describe SCP as a document for communication with HCP	I.3. Demonstrate intention to use the SCP to communicate with their HCP about questions and concerns	SE.3. Identify questions for their providers	A.3. Demonstrate positive attitude towards asking questions of providers	OE.3.a. Expects that SCP will help their communication with providers about questions and concerns OE.3.b. Demonstrate increased confidence to ask questions
**PO.4. Survivors will share SCP with family members**	K.4.a. Understand that sharing the SCP with family increases their sense of control over their treatment and cancer related issues K.4.b. Express the importance of sharing SCP with family	I.4. Demonstrate desire to share SCP with family members	SE.4. Express confidence to involve family members in survivorship care	A.4. Express positive attitude towards involving the family in their survivorship care	OE.4. Expect that involving and sharing the SCP with their family will help them better manage their survivorship care
**PO.5. Survivors will recognize/identify the areas of supportive care needs**	K.5. Recognize the importance of supportive care for survivorship	I.5. Demonstrate desire to identify their supportive care needs for survivorship	SE.5. Communicate their unmet supportive needs to their clinicians	A.5. Express positive attitude towards communicating their unmet supportive needs to their clinicians	OE.5. Expect that identifying and communicating their needs will receive support
**PO.7. Survivors will Seek instrumental support (e.g., transportation, translation, coordination, etc.)**	K.7. Describe the importance of seeking instrumental support	I.7.a. Demonstrate intention to seek instrumental support I.7.b. Demonstrate intention to communicate structural barriers	SE.7. Consult with patient navigator about their need for instrumental support	A.7. Express positive attitude towards expressing their need for instrumental support with patient navigator	OE.7. Expect that consulting with a patient navigator will help to overcome structural /organizational barriers
